# Dermatology services: The new normal post COVID‐19

**DOI:** 10.1002/ski2.30

**Published:** 2021-03-29

**Authors:** S. Yuen

**Affiliations:** ^1^ Blizzard Institute Barts and The London School of Medicine and Dentistry London UK

## Abstract

The article discusses the impact of COVID‐19 on the future of dermatology services. It will explore the changes dermatology services may need to follow to minimise disease transmission. This will include an integration of teledermatology into everyday practice, a shift in dermatology training, and change in dermoscopy technique.

## Graphical abstract

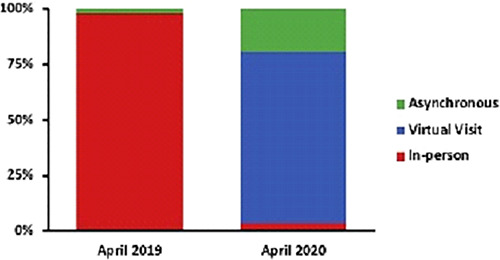

The article discusses the impact of COVID‐19 on the future of dermatology services. It will explore the changes dermatology services may need to follow to minimise disease transmission. This will include an integration of teledermatology into everyday practice, a shift in dermatology training, and changes in dermoscopy techniques.

## 1 INTRODUCTION

Drastic changes were imposed upon dermatology services throughout the coronavirus disease 2019 (COVID‐19) pandemic. In‐person consultation numbers dropped to single digits, whilst witnessing a threefold surge in the usage of teledermatology services.^1^ Despite the ongoing vaccine roll‐out worldwide, the uncertainty regarding its long‐term effectiveness and safety remains high. Therefore, it is crucial to consider the lasting impact that COVID‐19 will have for years to come and how dermatology services must alter their services to accommodate this change.

### 1.1 Teledermatology

**FIGURE 1:**
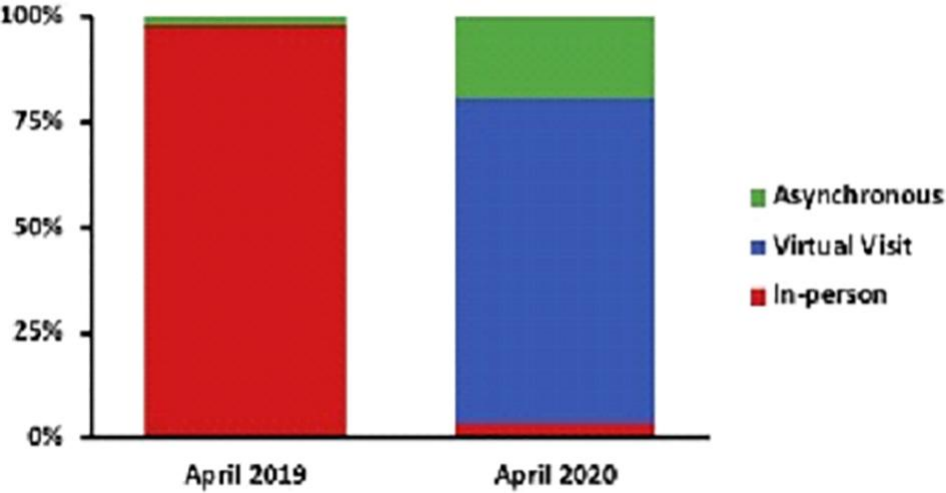
Rapid change in the proportion of each type of dermatology appointments offered to patients from April 2019 to April 2020

A key player in dermatology consultations has been the increased adoption of teledermatology services. This has been deemed as part of the digital revolution ignited by COVID‐19, dramatically expediting its acceptance and integration into everyday practice. Many practices faced the transition from having no teledermatology services prior to the pandemic, to using it almost exclusively within the span of a few months. This has been witnessed by a practice in Massachusetts, as highlighted in Figure 1.^2^

Teledermatology not only reduces the risk of COVID‐19 exposure by reducing face‐to‐face (F2F) interactions but also allows for a more streamlined triage hence cutting down waiting times. The flexibility of ‘store and forward’ (SAF) teledermatology allows for asynchronous dermatology reviews. This enhances continuity of care and increases accessibility for consultations which is particularly pertinent in areas that face health disparities in dermatology service provision. In the future, hospital admission may only be recommended in certain circumstances, such as for patients who require further assessment to confirm their diagnosis. Live video conferencing (LVC) offers another alternative that permits immediate physician–patient interaction.

However, a shift towards utilising teledermatology will have many pitfalls. Poor image quality and lack of communication can result in misdiagnosis. Technology associated health inequalities, such as the lack of appropriate software, which is more common in LVC, must not be overlooked. SAF helps to address these issues as sessions can be prepared without the Internet. Although SAF offers a higher diagnostic accuracy over LVC, a systematic review showed that LVC is superior when comparing consultation time effectiveness.^3^ Data security and encryption will also be paramount to ensuring the safe transfer of photographs between dermatologists and patients. Evidently, SAF and LVC both have their merits, and their application should be assessed on a case‐by‐case basis. It is important to highlight that teledermatology should not be a substitute for F2F but be used as a complementary service.

Teledermatology also offers a more cost‐effective approach compared to F2F consultations through actors such as reducing staff costs and loss of productivity. Therefore, this increases its appeal as the NHS continues to face financial pressures during its age of austerity, which has been further heightened by COVID‐19 that is likely to cost the health services an estimated extra £40bn a year.

**FIGURE 2:**
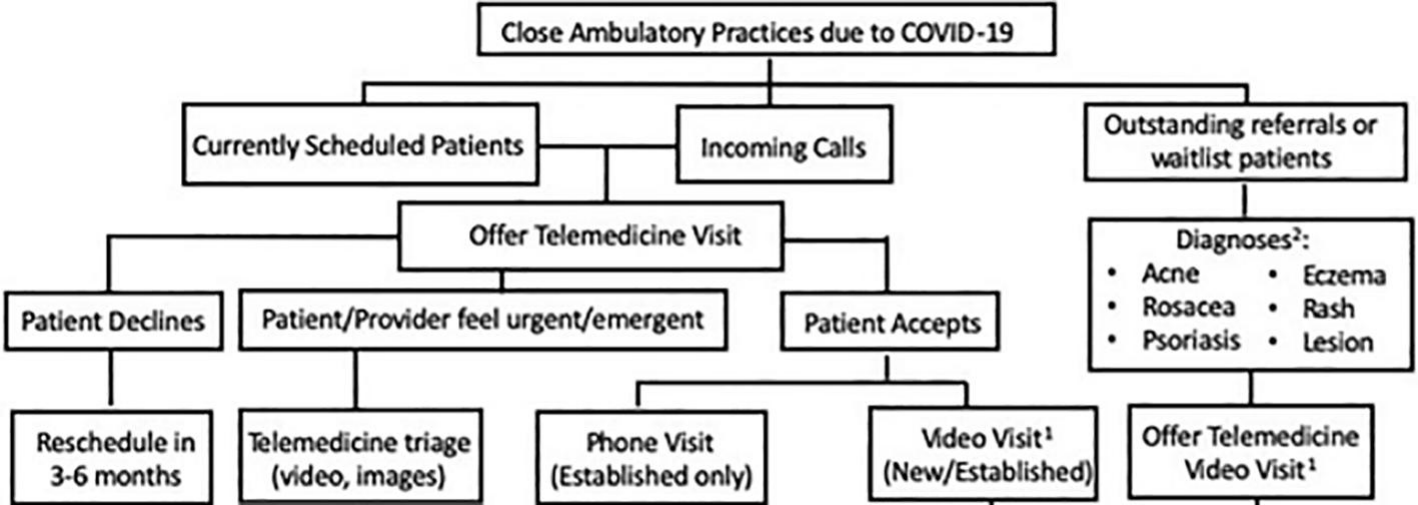
Teledermatology service algorithm for the screening of all dermatology patients

Figure 2 illustrates an algorithm for patient screening using teledermatology,^4^ that may be increasingly implemented worldwide to facilitate nonessential F2F minimisation.

### 1.2 Education

Teaching across all specialties has been greatly interrupted, from cancellations of conferences to suspension of practical examinations. Many dermatology trainees were redistributed to working in acute or general medicine. Webinars and virtual conferences will likely become the mainstay of dermatology training to curtail student gatherings whilst providing sufficient teaching to maintain the confidence and competency of future dermatologists.

Photographs collected through teledermatology consultations, with patient consent, offers an invaluable resource for dermatology trainees as diagnoses through assessing skin photographs will become increasingly prevalent.

### 1.3 Burnout

The increasing working hours to tackle the backlog of patients accumulated from the pandemic will undoubtedly lead to an increased risk of burnout, with technology‐associated fatigue becoming widespread. A survey revealed that more than 40% of doctors had experienced work‐related mental health issues during the pandemic.^5^ Asynchronous teledermatology may also lead to an increased overlap between personal and work life.

A systematic approach to promoting well‐being is vital as we begin to enter the ‘new‐normal’ post‐COVID to maintain provider and patient satisfaction and outcome. Possible solutions can include allocating protected time to pursue academic interests or scheduling time within the day for work to be done alone.

### 1.4 Dermoscopy

Dermoscopy plays a crucial role in the diagnosis of conditions such as melanomas. In efforts to reduce COVID‐19 spread, increased use of dermoscopy over biopsies may arise due to its noninvasive technique. However, dermoscopy has been suggested as being a potential spreader of nosocomial infections, such as human papillomavirus.^6^ Given the ability of COVID‐19 to remain viable on stainless steel for up to 72 h, safety measures must be taken to prevent viral spread between patients and dermatologists.^7^

Precautions to minimise viral spread include the use of polarised noncontact dermoscopy as direct contact with the lesion is not required.^8^ Another technique is the use of a disposable glass microscope slide between the lesion and the dermoscope. The dermoscope and the surfaces exposed to the patient should be disinfected, along with appropriate hand sanitisation before and after the consultation, with regular use of emollients to minimise occupational dermatitis. Exposure to mucous membranes during the examination should prompt the use of eye protection to minimise ocular transmission.^9^

Prior to each consultation, a thorough history should be taken. Albeit rare, a systemic review reported cutaneous manifestations of COVID‐19 to include erythematous maculopapular and vascular rashes.^10^ Training of dermatologists to recognise potential COVID‐19 presentations should be introduced to signpost any COVID‐suspected patients to appropriate services as quickly as possible. Dermatoscopic examinations must be delayed for 14 days if suggestive COVID‐19 symptoms are observed.

## 2 CONCLUSION

Many unprecedented challenges have been imposed on healthcare services upon the onset of the COVID‐19 pandemic. The continuously fluctuating trends in infection rates and the rising number of COVID‐19 variants render it difficult to predict when dermatology services will resume normality. The application of stringent measures for the foreseeable future is critical in minimising disease resurgence whilst ensuring that the clinical needs of patients are met with the best possible care.

## CONFLICTS OF INTEREST

The author declares that there are no conflicts of interest.

## Data Availability

Data sharing not applicable to this article as no datasets were generated or analysed during the current study.
